# Breaking microaggressions without breaking ourselves

**DOI:** 10.1007/s40037-019-0518-1

**Published:** 2019-05-28

**Authors:** Javeed Sukhera

**Affiliations:** Department of Psychiatry and Paediatrics, Schulich School of Medicine and Dentistry, Western University, London, Ontario Canada

Chester Middlebrook Pierce was an American psychiatrist and athlete who first coined the term microaggressions to describe brief indignities that convey hostility towards a social group [1]. In his research, Dr. Pierce emphasized the toxic effects of racism on communities and populations. He wrote about how seemingly innocuous actions can have deep and long-lasting effects on marginalized communities, and strove to promote more therapeutic relationships between those who find themselves advantaged, with those who are disadvantaged [2].

In a recent paper on microaggressions in Perspectives on Medical Education, Espaillat and colleagues found such indignities are commonplace within clinical learning environments. Microaggressions were perceived in both preclinical and clinical learning environments. While most students could recognize and identify microaggressions, the authors also found that 44% of respondents had not even heard of the term [3]. The undergraduate medical students surveyed defined microaggressions as passive-aggressive diminutive comments or actions that accumulate to have a significant impact. However, some participants contrasted with others. These individuals reacted to the term microaggressions with denial, defense, and indifference. One participant went far enough to state:

*Microaggressions are a way of perpetuating the culture of victimhood that plagues our society and polluting the minds of today’s over privileged, faux social justice warrior youth.*

The initial reaction to such statements may be anger. We may be tempted to reply with evidence of how microaggressions contribute to psychological distress, erode trust, and hurt our collective wellbeing [4–8]. We may want to explain that microaggressions exist on a continuum that includes mistreatment, harassment, racism, misogyny and discrimination. Some of us may react by invoking a concept related to microaggressions: gas lighting. When individuals experience discrimination or harassment in the workplace, gas lighting refers to when perpetrators of injustice lead victims to question their subjective reality. More commonly we do not react, we educate; we respond by addressing the problem we encounter through education and training.

Education related to microaggressions takes many shapes and forms. Health professionals are often educated about microaggressions through curricula that enhance awareness, communication skills, and promote feedback seeking [9], while addressing the intersecting forms of discrimination and prejudice within organizations [10]. Despite some promising results, efforts to promote education about microaggressions have been constrained by numerous barriers. For example, simply educating individuals to address broader structural inequities without attention to organizational and cultural influences may prove unsustainable [11]. In addition, topics such as microaggressions can trigger negative emotional reactions for health professionals [12], leading educational efforts to backfire.

When you share with health professionals that despite their best intentions, they may perpetuate discrimination, this suggestion will likely be met with discomfort. Feedback about microaggressions has the potential to remind health professionals that they are not superhuman. Rather, that they have flaws, vulnerabilities and shortcomings. This creates dissonance within an academic culture that often expects perfection. Constantly striving to manage microaggressions within a culture that rewards high achievement has the potential to produce psychological distress. Therefore, education to address microaggressions may not achieve intended goals, while resulting in unintended consequences.

In our research, we explored how teachers and learners manage the tensions that occur during the process of learning about implicit biases, which is a concept that is intrinsically related to microaggressions. We learned that recognizing implicit biases triggers compartmentalization between the ideal version of ourselves, and who we truly are. This work suggests that successfully managing implicit biases requires reconciling these tensions through striving for the ideal, while accepting the actual. Safe and supportive relationships between teachers and learners were also considered essential to enacting the process of ‘striving while accepting’ [13].

Effectively addressing microaggressions through education therefore requires shifting our frame. Rather than creating a learning environment where discussing microaggressions leads to feelings of shame or guilt, we must work to normalize the concept. Instructors should emphasize that microaggressions can be a normal product of our experience. Normalizing microaggressions can help foster self-compassion and self-forgiveness, as learners are encouraged to strive for the best version of themselves while accepting that they will always be flawed.

Yet our attention may return to the survey participant who associated microaggressions with victimhood. If I ask you to imagine what this participant may look like, or what gender this participant might be, what would be the image that comes to mind? We should all catch ourselves when it comes to our potential to perpetuate biases and microaggressions. Injustice is a virus, and we are all susceptible to being infected. We are all both victim and perpetrator, and none of us are immune. Therefore, change starts with looking in the mirror; not by pointing our finger.
